# Rural–urban disparities in birth interval among women of reproductive age in Nigeria

**DOI:** 10.1038/s41598-022-22142-y

**Published:** 2022-10-19

**Authors:** Anthony Ike Wegbom, Adetomi Bademosi, Clement Kevin Edet, Kinikanwo Innocent Green, Leesi Sapira-Ordu, Adeniyi Francis Fagbamigbe

**Affiliations:** 1 Department of Community Medicine, College of Medical Sciences, Rivers State University, Port Harcourt, Nigeria; 2 Department of Obstetrics and Gynaecology, University of Port Harcourt Teaching Hospital, Port Harcourt, Nigeria; 3 Department of Obstetrics and Gynaecology, College of Medical Sciences, Rivers State University, Port Harcourt, Nigeria; 4 Department of Epidemiology and Medical Statistics, Faculty of Public Health, College of Medicine, University of Ibadan, Ibadan, Nigeria

**Keywords:** Epidemiology, Risk factors

## Abstract

Nigeria like most developing nations still faced with a higher rate of short birth interval (SBI), and its associated consequences, such as adverse maternal and child health outcomes. This study aimed to determine the distribution and factors associated with SBI in rural and urban Nigeria. The data for this study were extracted from the 2018 Nigeria Demographic and Health Survey (2018 NDHS). Statistical analyses were descriptive analysis and binary logistic model. The proportions of SBI in rural and urban Nigeria were 20.7% and 20.3% respectively. Women’s age, geopolitical region, education level, and the number of children ever born were significantly associated with SBI in rural and urban Nigeria. Maternal Wealth index and antenatal care visits were only significant in rural while working status was only significant in urban Nigeria after controlling for other factors. Higher odds of SBI for middle class women than poor women (AOR = 1.19, 95% CI = 1.06–1.35), and increase in ANC visits reduces the odds of having SBI: 4–7 visits (AOR = 0.87, 95% CI = 0.77–0.98) and > 7visits (AOR = 0.83, 95% CI = 0.69–0.99). There were slight disparities in the prevalence of short birth intervals in rural and urban areas. Wealth index and ANC visits were only significant in rural Nigeria. Public health awareness campaigns should be strengthened to drive the importance of birth spacing techniques such as the utilization of modern contraceptives and breastfeeding in all the geo-political regions and across all age strata. Women particularly those residing in the rural areas should be encouraged to advance their education to at least a secondary level and enlightened on the importance of ANC.

## Introduction

Birth spacing, also known as an inter-birth interval (IBI), or inter-pregnancy interval refers to how soon after a prior pregnancy a woman becomes pregnant or gives birth again. Optimal birth spacing (defined as an inter-birth interval length of 24–59 months) is incontrovertibly linked to better health outcomes for both mothers and babies^1–3^. The World Health Organization (WHO) currently recommends an interval between the last live birth and the next pregnancy of at least 24 months^4^, and any birth less than this is considered a short birth interval (SBI). Short birth intervals lead to adverse maternal, perinatal, neonatal, and child health outcomes^5,6^. Short birth interval is associated with under-five mortality and low birth weight^7,8^. Globally, it is estimated that 25% of births still occur less than 24 months, and most of these births were observed in Central Asia (33%) and Sub-Saharan Africa (20%)^9^.

In Nigeria according to the 2018 Nigeria Demographic and Health Survey stood at 25% with a median birth interval of 30.9 months^10^. This suggests that Nigeria, like other developing nations, still is faced with a high rate of short birth intervals. Other African countries with similar rates include Rwanda: 20%, Uganda: 25.3%, Cameroon: 21.3% and Ethiopia: 24.9%, and these high rates were attributed to a lack of access to family planning options^11^. A study in Abuja, the capital city of Nigeria reported that 50% of women had birth to pregnancy intervals of fewer than 24 months, while 14 (3.5%) had birth to pregnancy intervals of greater than 60 months^1^. A similar study in Enugu, Nigeria also reported that the median inter-birth interval was 21.5 months^2^.

Few factors have been associated with birth intervals. Some of these are direct factors such as frequency of sexual activity, use of contraceptives, postpartum in fecundability, abortion, and sterility while indirect factors include socioeconomic and cultural factors^12,13^. Interbirth interval is also influenced by sociodemographic, economic, and birth history^6^. Fayehun et al. reported that the type of contraceptives used, living in rural or urban settings, wealth index, husbands’ occupation, and sex of preceding children determined birth intervals^14^. Having more female children and the last birth been a female significantly reduced birth intervals in the quest for a male child^14^. The use of contraceptives for birth spacing was also seen to be affected by male child preference where the last child is a female, a woman would like to use a short-acting contraceptive in order not to wait too long to get a male child^6^.

While adequate birth spacing is considered as a very significant factor for the health of women and their children^11^, inadequate birth spacing was rated as the highest risk faced by African women during pregnancy compared to other pregnancy-related outcomes^15^. Some studies in Nigeria and elsewhere have also shown a significant association between preceding birth intervals and under-five mortality^16–20^. However, studies on the determinants of the short birth interval using nationally representative data such as Nigeria Demographic and Health Survey (NDHS) are sparse in Nigeria. Existing studies were Hospital-based^2,21–23^, and each of them was only limited to the city or town in which the Hospital is located. This study bridged the gap by estimating the determinants of short birth intervals at the national level using NDHS data.

Given the above, it is important to gain a deeper understanding of the disparities existing among women living in rural and urban Nigerians in terms of interbirth interval. This study, therefore, aimed to determine the distribution and factors associated with short birth intervals in rural and urban Nigeria.

## Methods

### Data source

The data for this study were extracted from the 2018 Nigeria Demographic and Health Survey (NDHS) conducted by the National Population Commission (NPC) and ICF international. Being a national survey, it elicits information from women and men of reproductive age across the 36 states and the Federal Capital Territory (FCT) Abuja, nominated through a stratified two-stage cluster sampling technique. The NDHS, like other Demographic and Health Surveys (DHS) is conducted every five years in the consenting low- and middle-income countries.

### Data

The data involved 41,821 women residing in rural and urban Nigeria, extracted from women data of 2018 NDHS.

### Study variables

The outcome variable in this study was preceding birth interval which was recoded as < 24 months and ≥ 24 months. This is based on the definition of the World Health Organization^4^. The explanatory variables were sociodemographic and health care-seeking characteristics.

The explanatory variables and their categories were:

Mother’s age: < 20/20–29/30–39/ ≥ 40 years.

Child’s sex: Male/female.

Mother’s Religion: Christianity/Islam/Others.

Current Region of residence: North-central/North-east/ North-west/South-east/South-south/South-west.

Current place of residence: Urban/rural.

Mother’s educational level: None/primary/secondary/higher.

Marital status: Never married/married.

Employment status: Unemployed/employed.

Wealth index: Poor/middle/rich.

Number of children ever born: 0–2/ 3–4/ > 4.

Antenatal care visits: None/1–3/4–7/ > 7 visits.

### Data analysis and statistical technique

We analyzed the data using univariate, bivariate and multivariate techniques. At the univariate level, proportion was used to describe the characteristics of the respondents; at the bivariate technique level, chi-square was utilized to assess the association between the characteristics and the birth interval; while binary logistic regression was fitted at the multivariate level to determine the influence of these factors on birth interval to explain the disparities in the birth interval between rural and urban women.

The outcome variable, the birth interval, was categorized and coded as (≥ 24 months = 0 and < 24 months = 1) and used in the logistic regression. Firstly, we described the characteristics. Secondly, we assessed the association between the explanatory variables and the birth interval; and lastly, only the significant variables were included in the multiple binary logistic regression model. Three logistic regression models were fitted for the study: the entire country, rural Nigeria, and urban Nigeria. To account for the complex nature of the DHS data, we adjusted for sampling weight, clustering, and stratification. The measure of associations was the odds ratio, and its statistical significance was assessed at 95% confidence interval excluding unit and *P*-value < 0.05. Analysis was arried out using Stata version 15.0 (StataCorp, College Station, TX)s.

The logistic regression model is expressed as:

1 $$In\left[ {\frac{p}{1 - p}} \right] = \beta_{0} + \mathop \sum \limits_{i = 1}^{k} \beta_{i} X_{i}$$

where *p* is the probability of an event to occur (having a short birth interval) and 1-*p* is the probability of the event not occurring (not having a short birth interval), $\frac{p}{1 - p}$ is the “odds” of occurrence of the event against its non-occurrence. $\beta_{i}$ and $X_{i}$ are the regression coefficient of the explanatory variables and the explanatory variables (sociodemographic factors) respectively; and $\beta_{0}$ is the constant for the logistic regression model.

All methods were carried out in accordance with relevant guidelines and regulations. In addition, all experimental protocols and ethical approval were obtained from the National Health Research. Ethics Committee of Nigeria (NHREC) (ref. no. NHREC/01/01/2017). Written informed consent was obtained from all subjects and/or their legal guardian(s).

## Results

**Figure 1:**
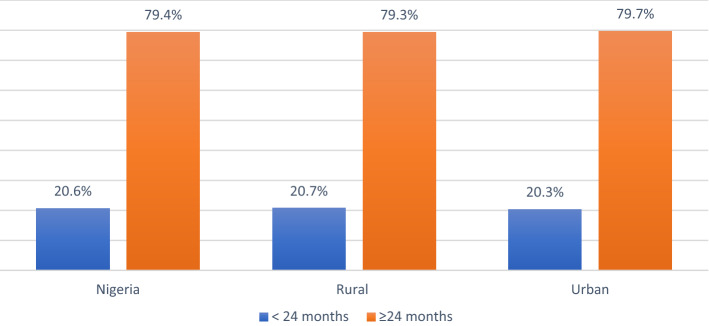
Disparities in proportion of birth interval in Nigeria.

The distributions of the characteristics and their association with birth interval are shown in Table 1. The results show that all factors are significantly associated with short birth intervals across Nigeria (*P* < 0.05), except the sex of the child (*P* = 0.749), and so was excluded in further analysis. The proportion of < 24 months and ≥ 24 months birth intervals were respectively 20.6% vs 79.4% for Nigeria, 20.3% vs 79.7% for urban Nigeria and 20.7% vs 79.3% for rural Nigeria, as shown in Fig. 1.

**Table 1:** Characteristics and Association of respondents with Birth Interval in Nigeria.

Characteristics	≥ 24 months number (%)	< 24 months number (%)	Chi-square	*P*-value
Overall	20,084 (79.4)	5196 (20.6)		
**Place of residence**			27.59	0.000
Rural	12,625 (79.3)	3299 (20.7)		
Urban	7459 (79.7%)	1897 (20.3)		
**Maternal age (years)**			2155.2	0.000
< 20	156 (65.0)	84 (35.0)		
20–29	5862 (75.7)	1878 (24.3)		
30–39	8008 (80.9)	1896 (19.1)		
≥ 40	6058 (81.9)	1338 (18.1)		
**Child sex**				
Male	7303 (56.6)	5609 (43.4)	0.1	0.749
Female	7020 (56.8)	5348 (43.2)		
**Religion**			253.9	0.014
Christianity	8919 (79.7)	2269 (20.3)		
Islam	10,990 (79.3)	2869 (20.7)		
Others	175 (75.1)	58 (24.9)		
**Region**			861.6	0.000
North Central	3770 (82.2)	816 (17.8)		
North East	3810 (78.0)	1074 (22.0)		
North West	5340 (78.5)	1462 (21.5)		
South East	2242 (75.0)	747 (25.0)		
South South	2191 (78.2)	610 (21.8)		
South West	2731 (84.9)	487 (15.1)		
**Educational level**			209.1	0.004
No education	8759 (78.9)	2345 (21.1)		
Primary	3915 (81.3)	900 (18.7)		
Secondary	5808 (79.0)	1542 (21.0)		
Higher	1602 (79.7)	409 (20.3)		
**Marital status**			29.5	0.018
Not married	18,392 (79.3)	4811 (20.7)		
Married	1692 (81.5)	385 (18.5)		
**Employment status**			81.1	0.000
Unemployed	2534 (38.6)	4030 (61.4)		
Employed	8423 (45)	10,293 (55)		
**Wealth index**			83.9	0.002
Poor	8684 (79.1)	2295 (20.9)		
Middle	4259 (79.1)	1123 (20.9)		
Rich	7141 (80.1)	1778 (19.9)		
**Number of children ever born**			683.1	0.001
0–2	3685 (78.5)	1011 (21.5)		
3–4	6798 (80.0)	1695 (20.0)		
> 4	9601 (79.4)	2490 (20.6)		
**Antenatal care visits**			13.1	0.002
None	3674 (79.0)	975 (21.0)		
1–3	2490 (77.9)	705 (22.1)		
4–7	5414 (80.5)	1309 (19.5)		
> 7	2820 (81.2)	652 (18.8)		

*Statistically significant at *P* < 0.05.

Table 2 shows the results of the analysis of association between birth interval and the women characteristics using logistic regression. The result indicates the impact of sociodemographic and health care related factors contributing to short birth interval in rural and urban Nigeria. The result indicated higher odds of short birth interval in rural areas (AOR = 1.16, 95% CI = 0.96–1.16) compared to urban areas, though not statistically significant. Analysis showed a significant decrease in odds of short birth interval with increased maternal age in Nigeria (20–29 years: AOR = 0.40, 95% CI = 0.34–0.47; 30–39 years: AOR = 0.22, 95% CI = 0.19–0.27; ≥ 40 years: AOR = 0.15, 95% CI = 0.11–0.19); and rural Nigeria (20–29 years: AOR = 0.39, 95% CI = 0.33–0.47; 30–39 years: AOR = 0.24, 95% CI = 0.19–0.30; ≥ 40 years: AOR = 0.15, 95% CI = 0.11–0.21) and urban areas (20–29 years: AOR = 0.37, 95% CI = 0.27–0.51; 30–39 years: AOR = 0.17, 95% CI = 0.11–0.23; ≥ 40 years: AOR = 0.10, 95% CI = 0.07–0.17).

**Table 2:** Factors associated with short birth interval in Nigeria, rural and urban Nigeria.

Factors	Nigeria			Rural Nigeria			Urban Nigeria		
	COR	AOR	95% CI for AOR	COR	AOR	95% CI for AOR	COR	AOR	95% CI for AOR
**Place of residence**									
Urban	1.00	1.00							
Rural	1.03	1.12	0.96–1.16						
**Maternal age (years)**									
< 20	1.00	1.00		1.00	1.00		1.00	1.00	
20–29	0.52*	0.40*	0.34–0.47	0.53*	0.39*	0.33–0.47	0.49*	0.37*	0.27–0.51
30–39	0.36*	0.22*	0.19–0.27	0.38*	0.24*	0.19–0.30	0.32*	0.17*	0.11–0.23
≥ 40	0.24*	0.14*	0.11–0.19	0.25*	0.15*	0.11–0.21	0.20*	0.10*	0.07–0.17
**Religion**									
Christianity	1.00	1.00			1.00		1.00	1.00	
Islam	1.26*	1.06	0.91–1.18	1.35*	1.15	0.97–1.35	1.10*	0.91	0.73–1.12
Others	1.15	0.91	0.52–1.27	1.25*	1.04	0.64–1.69	0.95	0.67	0.29–1.53
**Region**									
Northcentral	1.00	1.00		1.00	1.00		1.00	1.00	
Northeast	1.42*	1.15*	1.02–1.33	1.40*	1.09	0.93–1.29	1.49*	1.40*	1.09–1.78
Northwest	1.52*	1.08	0.97–1.26	1.53*	1.09	0.93–1.28	1.49*	1.04	0.83–1.31
Southeast	1.43*	1.95*	1.59–2.23	1.46*	2.09*	1.66–2.63	1.44*	1.75*	1.36–2.26
Southsouth	1.12*	1.48*	1.27–1.79	1.07	1.42*	1.15–1.76	1.26*	1.56*	1.16–2.08
Southwest	0.80*	0.82*	0.67–0.95	0.82*	0.90	0.69–1.17	0.80*	0.75*	0.58–0.95
**Educational level**									
Higher	1.00	1.00			1.00		1.00	1.00	
Secondary	0.83*	0.88*	0.78–0.99	0.77*	0.90	0.77–1.05	0.78	0.97	0.76–1.25
Primary	0.97*	1.06	0.97–1.29	0.83*	1.07	0.92–1.26	1.19*	1.24	0.97–1.57
No education	1.63*	1.45*	1.25–1.89	1.64*	1.58*	1.17–2.08	1.34*	1.61*	1.19–2.19
**Marital status**									
Never married	1.00				1.00		1.00	1.00	
Married	1.16*	1.11	0.92–1.34	1.23*	0.99	0.89–1.10	1.21*	1.26	0.94–1.69
**Employment status**									
Unemployed	1.00			1.00			1.00		
Employed	0.77	0.73*	0.68–0.80	0.78*	0.95	0.87–1.03	0.76*	0.94*	0.76–0.98
**Wealth index**									
Poor	1.00	1.00		1.00	1.00		1.00	1.00	
Middle	0.92*	1.12*	1.01–1.24	0.92*	1.19*	1.06–1.35	0.95	0.94	0.74–1.18
Rich	0.86*	1.13*	1.03–1.33	0.86*	1.12	0.95–1.32	0.89*	1.08	0.86–1.35
**Number of children ever born**									
0–2	1.00	1.00		1.00	1.00		1.00	1.00	
3–4	0.76*	0.69	0.58–1.05	0.45*	0.21*	0.03–0.37	0.83*	0.96	0.81–1.13
> 4	0.80*	0.82*	0.77–0.96	0.73*	0.67*	0.42–0.96	0.65*	0.24*	0.22–0.29
**Antenatal care visits**									
None	1.00	1.00		1.00	1.00		1.00	1.00	
1–3	1.07	1.03	0.92–1.15	1.03	1.02	0.89–1.16	1.15	1.06	0.82–1.36
4–7	0.91*	0.87*	0.78–0.96	0.89*	0.87*	0.77–0.98	0.95	0.87	0.69–1.08
> 7	0.87*	0.86*	0.75–0.98	0.88	0.83*	0.69–0.99	0.86	0.87	0.68–1.11

*Statistically significant at *P* < 0.05.

There was significantly higher odds of having a short birth interval among rural women living in only the South-east (AOR = 2.09, 95% CI = 1.66–2.63) and South-south (AOR = 1.42, 95% CI = 1.15–1.76). While among women residing in the urban areas, living in the North-east (AOR = 1.41, 95% CI = 1.09–1.78), South-east (AOR = 1.75, 95% CI = 1.36–2.26) and South-south (AOR = 1.56, 95% CI = 1.16–2.08) were significantly more likely to have short birth interval compared with those living in the North-central; whereas living in south-west urban Nigeria was significantly less likely to have short birth interval compared with those living in the North-central (AOR = 0.75, 95% CI = 0.58–0.95).

Whereas women who had no formal education were 61% and 58% significantly higher odds of having short birth interval than women with higher education in urban Nigeria (AOR = 1.61, 95% CI = 1.19–2.19) and rural Nigeria (AOR = 1.58, 95% CI = 1.17–2.08) respectively. Furthermore, employed women residing in the rural areas had lower odds of having short birth interval than unemployed women (AOR = 0.95, 95% CI = 0.87–1.03), though not statistically significant. Whereas, in urban areas employed women had significantly lower odds of having short birth interval than unemployed women (AOR = 0.74, 95% CI = 0.67–0.84). Regarding the household wealth index, middle wealth index women living in rural areas were 1.19 times more likely to have short birth intervals compared to poor women (AOR = 1.19, 95% CI = 1.06–1.35). But in the urban areas, middle wealth index women had a lower odds of having short birth intervals than the poor women, though not statistically significant (AOR = 0.94, 95% CI = 0.74–1.18).

Women in rural Nigeria who have had three to four deliveries and above were less likely to have a short birth interval (AOR = 0.21, 95% CI = 0.03–0.37) and (AOR = 0.67, 95% CI = 0.42–0.96) respectively than those with two deliveries or less. In urban Nigerian only women who had more than 4 children were significantly less likely to have a short birth interval than women who had two children or fewer (AOR = 0.24, 95% CI = 0.22–0.29). But those who had 3–4 children had lower odds of having short birth intervals compared with women who had two children or less, though not statistically significant. Women who attended antenatal care at least four times during pregnancy had lower odds of having short birth intervals in only rural Nigeria than those who did not attend: Four to seven ANC visits (AOR = 0.87, 95% CI = 0.77–0.98) and more than seven ANC visits (AOR = 0.83, 95% CI = 0.69–0.99).

## Discussion

This study was designed to assess short birth interval and its determinant with a focus on rural–urban variation in Nigeria. We defined a short birth interval as having another birth before 24 months of having a preceding birth. The overall prevalence of short birth intervals in Nigeria was 20.6%. While rural and urban areas of Nigeria were 20.7% and 20.3% respectively. This result is higher than the SBI reported in Rwanda, but lower compared to findings in some other African countries^5,11^ as well as in Brazil^12^. The observed differences could be attributed to the prevalent socio-geographical and cultural differences in the studied areas. These results indeed call for urgent action considering the adverse consequences of a short birth interval to mothers and children. Though all the variables were significantly associated with short birth intervals at the bivariate level, the multivariate analysis showed that maternal age at childbirth, geopolitical region, number of children ever born, and ANC visits were all statistically associated with short birth intervals. Although we found the sex of the child not be significant in the current study, the quest for male children or children in the Africa context, Nigeria inclusive, could affect birth interval among women.

While the educational level was significant in urban areas, the household wealth index was significantly associated with short birth intervals in rural areas at a multivariate level. Though, both variables were significant in Nigeria.

This study showed that the odds of having a short birth interval decrease as the mother’s age at birth of the child increases. In the other words, the odds of having the next child within 24 months is reduced as women get older, and this result agreed with previous studies^3,24^. Fecundity could be a possible explanation for this, as younger women are highly fecund than older women and the likelihood of younger women using contraceptives is less compared to older women because they have a high desire to bear children^24^. More so, younger women are likely to be less privileged and as such may not afford more birth spacing techniques compared to older women as shown in a study conducted in Finland^25^.

The study revealed that women residing in rural Nigeria were more likely to have short birth intervals compared with those residents in urban areas. This again substantiates the essence of this study, as it is believed that contributing factors to short birth interval is dependent on the place of residence, and as such could not be the same. This is consistent with reports of similar studies which indicate a relatively higher occurrence of short birth intervals in rural areas compared to urban areas^14,26,27^. This could be attributed to the relatively lower information on birth intervals and the nonavailability of modern contraceptives in rural areas compared to urban areas. Women who have information about short birth intervals through any media channel are expected to have a better understanding of the negative impact of short birth intervals on maternal and children’s health. As a result, women who have no exposure to any media are more likely to experience short birth intervals than those who have media exposure^28,29^.

Whereas all the zones in the country except North-west were associated with high odds of having a short birth interval at the national level and rural areas, only south-east and south-south were significantly associated with high odds of having short birth interval among urban residents. This could be because of the under-utilization of contraceptives in all the regions in rural and urban Nigeria by both men and women^30^.

The study also revealed that women that attended the WHO minimum of four antenatal care visits were less likely to have a short birth interval than those that attended less than the minimum visits in rural Nigeria. This is because ANC attendees have access to health education that explains the consequences of short birth intervals and information and materials that will enable them to prolong pregnancy to their desired time. Having more than two children was less likely to have a short birth interval than having less for women residing in both rural and urban areas of Nigeria. This is true because these women that had more children may have achieved their desired family size and may feel less pressure or be in less of a hurry to get pregnant again^3^.

This study has observed that Maternal education is protective of short birth intervals as an increase in educational attainment leads to a decrease in the likelihood of experiencing short birth intervals. This implies that educated women were more likely to have spaced births than non-educated women. This finding is consistent with other studies in Ethiopia and Pakistan^31–33^. The explanation might be attributed to the fact that better-educated women are more likely to use contraception to delay their inter-birth intervals and may also be exposed to more health education. Furthermore, there is a likelihood that literate women may be engaged in careers that are not well-suited to childbearing^32,34^.

The household wealth index was also identified as a significant determinant of short birth interval in rural Nigeria. This study revealed that the odds of experiencing short birth intervals were less common in households in the rich and middle wealth index than in the poor wealth index. This is consistent with studies carried out in Ethiopia, Saudi Arabia^31,35^. The possible explanation for this could be that privileged women may have more access to health care education and information and could afford health care services that apply logically enhanced short interbirth interval^31^. Furthermore, there was a disparity in the association between working status and short birth in rural and urban Nigeria. Whereas in urban areas, working-class women had significantly lower odds of having short birth interval, it was not significant in rural areas. The insignificance of working status in rural areas could be that in rural Nigeria it is difficult to distinguish between working and non-working women because all women are involved in one activity to other. The significant association between employment status to short birth interval found in this study was consistent with other studies done abroad^36,37^. The reason has been that working-class women can afford modern contraceptives that could be used to delay pregnancy to enable them to put in some months in their job after maternity leave. Married women have higher odds of experiencing short birth intervals than never-married women. The reason may be that married women are less likely to use contraceptives than never-married women^38^.

The major strength of this study was the fact that it utilized nationally representative data collected through standardized questionnaires and valid sampling procedures. While the limitation was on the ground that the design was cross-sectional which involves some missing information on reproductive indicators. Our findings should be interpreted with caution as we could not establish causation.

## Conclusion

This study has demonstrated disparities in short birth interval in rural and urban Nigeria, by estimating prevalence and identifying factors influencing short birth intervals in these areas. There were slight disparities in the prevalence of short birth intervals in rural and urban areas. This is evident in the contributing factors as almost all the same factors such as maternal age at childbirth, geopolitical regions, educational attainment, and the number of children ever born were significantly associated with short birth intervals in both areas. It only differed in wealth index and the ANC visits for rural areas. Thus, policymakers and the relevant stakeholders should intensify existing strategies geared toward maintaining optimal birth spacing to ensure the well-being of mothers and children in rural and urban areas of Nigeria. Public health awareness campaigns should be strengthened to drive the importance of birth spacing techniques such as the utilization of modern contraceptives and breastfeeding in all the geo-political regions and across age strata. Finally, women, particularly in rural areas should be encouraged to advance their education to at least a secondary level and enlightened on the importance of ANC.

## Data Availability

The dataset used and analyzed during the current study is available from the corresponding author on reasonable request.
